# Nationwide Trends in Incidence of Stroke and Transient Ischemic Attack in Younger and Older Adults in Norway 2001 to 2021

**DOI:** 10.1161/JAHA.125.041029

**Published:** 2025-12-11

**Authors:** Elisabeth Kvalvaag, Vidar Hjellvik, Lars Kjerpeseth, Jannicke Igland, Hanne Ellekjær, Gerhard Sulo, Mariam Anjum, Kjersti S. Rabanal, Trygve Berge, Inger Ariansen

**Affiliations:** ^1^ Department of Chronic Diseases Norwegian Institute of Public Health Oslo Norway; ^2^ Department of Global Public Health and Primary Care University of Bergen Norway; ^3^ Department of Neuroscience Norwegian University of Science and Technology Trondheim Norway; ^4^ Stroke Unit, Department of Internal Medicine St. Olav’s Hospital Trondheim Norway; ^5^ Department of Medical Research Bærum Hospital, Vestre Viken Hospital Trust Gjettum Norway; ^6^ Department of Public Health University of Stavanger Norway; ^7^ Department of Cardiology Oslo University Hospital Ullevål Oslo Norway

**Keywords:** incidence, stroke, TIA, time trend, transient ischemic attack, Cerebrovascular Disease/Stroke, Ischemic Stroke, Intracranial Hemorrhage, Transient Ischemic Attack (TIA)

## Abstract

**Background:**

There is conflicting evidence on the time trends of stroke incidence in younger adults. We aimed to explore time trends in the incidence of stroke, stroke subtypes, and transient ischemic attack in different age groups in Norway the past 2 decades.

**Methods:**

In this nationwide registry‐based cohort study, we identified incident stroke cases from hospitalizations with stroke (ischemic stroke, intracerebral hemorrhage and unspecified stroke), transient ischemic attack, or out‐of‐hospital deaths due to stroke. Included individuals were aged ≥15 years with no prior hospitalization for stroke during the past 7 years. Incidence per 100 000 population was age‐standardized and presented by year, sex, and age group. Average annual percentage changes in incidence were estimated using weighted log‐linear regression.

**Results:**

From 2001 to 2021, we identified 229 857 incident total stroke cases (49.2% women) among 5 691 495 individuals in the Norwegian adult population. Incidence per 100 000 population decreased over time for all stroke subtypes in both men and women. However, the incidence of ischemic stroke for men aged <35 years increased annually by 1.6% (95% CI, 0.3–2.8). For intracerebral hemorrhage, the incidence was stable in younger men and women and the oldest women. For transient ischemic attack, both sexes experienced a sharp rise in the incidence until 2012, followed by a substantial decrease.

**Conclusions:**

Total stroke incidence declined from 2001 to 2021. However, less favorable time trends were observed in younger age groups, with a worrisome increase in ischemic stroke incidence among men aged 15 to 34 years. These trends are concerning and need further investigation.

## Graphical abstract

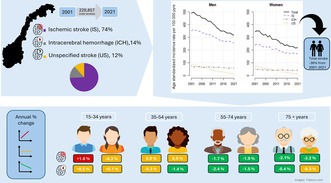

**Nonstandard Abbreviations and Acronyms**
AFNOR: Atrial Fibrillation in Norway
GBD: Global Burden of Disease
INTERSTROKE: Risk Factors for Ischemic and Intracerebral Hemorrhagic Stroke in 22 Countries

**Research Perspective**

**What Is New?**

- In this nationwide cohort study from Norway, age‐standardized incidence per 100 000 population decreased for total stroke and all stroke subtypes (ischemic, hemorrhagic, and unspecified stroke) from 2001 to 2021.
- Younger age groups exhibited less favorable trends than older age groups, with a concerning increase in ischemic stroke incidence among men aged 15 to 34 years.

**What Question Should Be Addressed Next?**

- What are the underlying factors behind rising ischemic stroke incidence among younger individuals, and how can targeted prevention strategies and early interventions be developed to address the risk profiles of younger individuals at risk for stroke?

More than 1 in 5 adults in Western Europe are estimated to experience acute stroke during their lifetime.^1^ Estimates from the GBD (Global Burden of Disease) study have shown a decline in the age‐standardized incidence of total stroke, ischemic stroke, and intracerebral hemorrhage (of 1.4%, 1.6%, and 1.5% per year, respectively) in Western Europe during 1990 to 2019.^2^ These modeled estimates align with pooled data from population‐based stroke incidence studies in high‐income countries.^3^ However, compared with other high‐income countries and Western Europe, Norway appears to be less successful in reducing stroke incidence.^2^, ^4^ Furthermore, diverging trends in stroke incidence have been reported from register‐based studies in the other Nordic countries.^5^, ^6^, ^7^, ^8^, ^9^, ^10^ In addition, there has been a substantial increase in the use of oral anticoagulants in recent years,^11^, ^12^ which may influence stroke subtype distributions. More specifically, the use of oral anticoagulants could lower the incidence of ischemic stroke while potentially increasing the risk of intracerebral hemorrhage.^9^, ^13^

Stroke death in Norway has declined since the 1970s.^14^ Possible explanations for decreasing stroke death are improved treatment options both in the acute and long‐term stages after stroke, primary and secondary prevention strategies, and a decline in stroke incidence.^15^, ^16^, ^17^, ^18^, ^19^ In support of the latter, the number of patients hospitalized with stroke in Norway decreased between 2010 and 2015.^20^ However, concerning trends have been observed in younger individuals (aged <50–55 years) with studies from Norway and other Western European countries reporting an increase in the incidence of stroke in this age group.^6^, ^21^, ^22^, ^23^, ^24^ On the other hand, other studies have suggested that stroke incidence in younger age groups may be stable or even decreasing.^7^, ^25^ A recent systematic review and meta‐analysis emphasized the need for continued monitoring of age‐specific trends in stroke incidence.^26^

There is a need for studies based on individual‐level data rather than modeled estimates. In Norway, updated nationwide data on incidence of stroke and transient ischemic attack (TIA) across different age groups are lacking. This study aimed to describe nationwide trends in incidence per 100 000 population of hospitalizations and out‐of‐hospital deaths due to ischemic stroke, intracerebral hemorrhage, and unspecified stroke and of hospitalizations with TIA in Norway during 2001 to 2021, overall and by sex and age group.

## Methods

### Data Linkage

This nationwide register‐based cohort study used data from the AFNOR (Atrial Fibrillation in Norway) study, which links individual‐level data from several population‐based registries in Norway. These include the Norwegian Patient Registry, the Norwegian Cause of Death Registry, a historic hospitalization database on cardiovascular diseases from the Cardiovascular Disease in Norway research project,^27^ and the National Population Register. Data from these sources were linked by a third party using the national unique personal identification number. Data from the Norwegian Patient Registry and the historic hospitalization database provided primary and secondary hospital discharge diagnoses for inpatients, coded according to the *International Classification of Diseases*, *Ninth Revision* (mainly used in the period 1994–1996) and *Tenth Revision* (mainly used from 1996 and forward) (*ICD‐9/10*).

The study was approved by the Norwegian Regional Committee for Medical and Health Research Ethics, Health Region South‐East (September 8, 2020; reference number 82710). The study complies with the Declaration of Helsinki and followed the Strengthening the Reporting of Observational Studies in Epidemiology guidelines.^28^ This research was done using data from health registries without patient involvement, and patient informed consent was not required.

The data that support the findings of this study are available from the Norwegian Health Data Authority and Statistics Norway, but restrictions apply to the availability of these data, which were used under license for the current study, and so are not publicly available. However, data and the methods used for the analyses are available from the authors upon reasonable request and with permission of the Norwegian Health Data Authority and Statistics Norway.

### Definition of Incident Stroke and TIA

An incident event of total stroke (*ICD‐9*: 431, 433, 434, 436; *ICD‐10*: I61, I63 [except I63.6], I64), ischemic stroke (*ICD‐9*: 433, 434; *ICD‐10*: I63 [except I63.6]), intracerebral hemorrhage (*ICD‐9*: 431; *ICD‐10*: I61), or unspecified stroke (*ICD‐9*: 436; *ICD‐10*: I64) was defined as an inpatient hospitalization with stroke as the main or secondary diagnosis or out‐of‐hospital death with stroke as the underlying cause, in both cases with no previous hospitalization for stroke in the past 7 years. (For ischemic and unspecified stroke, there was no hospitalization for ischemic or unspecified stroke in the past 7 years. For intracerebral hemorrhage, there was no hospitalization for intracerebral hemorrhage in the past 7 years. For total stroke, there was no hospitalization for any of the 3 in the past 7 years.) Stroke as a secondary diagnosis was not included if the primary diagnosis was previous stroke (*ICD‐9*: 438; *ICD‐10*: I69) or rehabilitation (*ICD‐9*: V57; *ICD‐10*: Z50.80, Z50.89).

An incident event of TIA was defined as a somatic inpatient hospitalization with a main or secondary diagnosis of TIA (*ICD‐9*: 435; *ICD‐10*: G45) with no previous hospitalization for ischemic stroke, unspecified stroke, or TIA in the past 7 years. TIA as a secondary diagnosis was not included if the primary diagnosis was previous stroke (*ICD‐9*: 438; *ICD‐10*: I69) or rehabilitation (*ICD‐9*: V57; *ICD‐10*: Z50.80, Z50.89).

### Study Design and Study Population

This nationwide cohort study included all individuals registered as residents in Norway and aged ≥15 years as of January 1 each year from 2001 to 2021. We calculated stroke incidence per 100 000 population where the numerator consisted of the number of incident cases of inpatient stroke hospitalizations and out‐of‐hospital deaths from stroke each year. The denominator for a given calendar year was the Norwegian population free of stroke (no previous hospitalization for stroke in the past 7 calendar years) as of January 1 that year. Because the majority of the population constituting the denominator for a given year did not die, emigrate, or have a stroke during the year, the incidence per 100 000 population can be considered a reasonable approximation of the incidence rate, except potentially among the oldest age groups, where deviations may be more pronounced.

### Statistical Analysis

The number of stroke cases, proportion of stroke subtypes, and incidence per 100 000 population are presented by sex and the following age groups: 15 to 34, 35 to 54, 55 to 74, and ≥75 years. A subgroup summary for the older population (aged 75–84 and ≥85 years) is presented in Figure S1. We used a fixed lookback period of 7 years to ensure consistent classification of stroke cases over time. The individuals defined with an incident stroke in 2001 had no stroke between dd.mm.1994 and dd.mm.2001, where dd.mm is the date of the stroke in 2001. Similarly, the individuals defined with an incident stroke in 2002 had no stroke between dd.mm.1995 and dd.mm.2002.

For each age group, age‐standardized incidence per 100 000 population with 95% CIs were calculated by direct standardization to the European standard population 2013 in 5‐year strata, using the ageadjust.direct function in the R‐package epitools (R Foundation for Statistical Computing, Vienna, Austria).^29^ Annual relative changes in incidence were estimated using weighted log‐linear regression. Each calendar year was assigned a weight corresponding to the inverse of the variance of the age‐adjusted incidence estimate for that year. Thus, years with more precise incidence estimates (ie, lower variance) contributed more strongly to the regression analysis. The annual percentage change in incidence was derived as (exp(β)−1)×100, where β represents the yearly increase in the logarithm of the incidence estimate. More details including the syntax are provided in the Supplemental Methods section. Separate regressions were conducted for the past 10 years, due to the inverted U shape of the TIA incidence peaking around 2012. These are presented in Figure S2.

Definition of education, income and country background, and methods for comparisons between incident total patients with stroke and the general Norwegian population are provided in Figure S3.

As a sensitivity analysis, we estimated 95% CIs for the annual percentage change by taking the autocorrelation in the residuals between adjacent years into account. More details are provided in the Supplemental Methods section, and the results are presented in Figure S4.

Statistical analyses were performed using R version 4.2.2.

## Results

From 2001 to 2021, we identified 229 857 incident total stroke cases (49.2% women) among 5 691 495 individuals in the Norwegian population aged ≥15 years, accounting for 82 820 703 person‐years. Of these, 74.4% were ischemic stroke, 13.9% were intracerebral hemorrhage, and 11.7% were classified as unspecified stroke. During the same period, we identified 76 533 incident TIA cases (51.0% women). Of the incident total stroke cases, 17 469 (7.6%) were out‐of‐hospital deaths (66.1% women) and 212 388 (92.4%) were inpatient hospitalizations (47.8% women). During the study period, the mean age at incident stroke decreased from 73.3 to 71.8 years in men and from 78.4 to 76.8 years in women. Stroke cases with stroke as a secondary diagnosis accounted for 22.5% of all stroke hospitalizations. Of these, 51.0% were ischemic strokes, 13.3% hemorrhagic strokes, and 35.7% were unspecified strokes.

**Figure 1:**
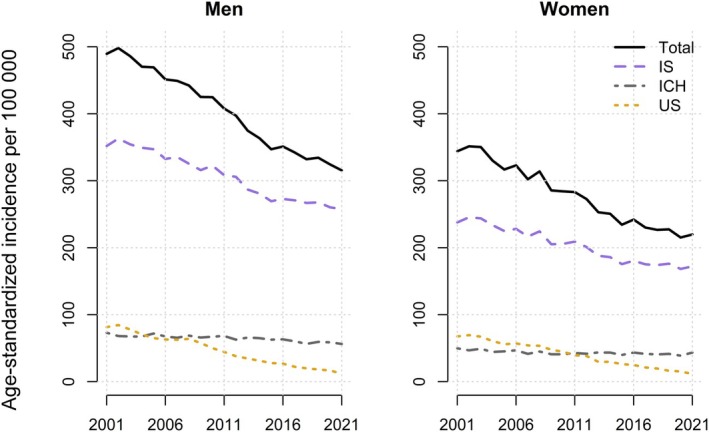
Age‐standardized incidence per 100 000 population for total stroke and stroke subgroups in men and women in Norway, 2001 to 2021. ICH indicates intracerebral hemorrhage; IS, ischemic stroke; and US, unspecified stroke.

The age‐standardized incidence per 100 000 population of total stroke decreased annually by 2.4% (95% CI, 2.3–2.6) in men and 2.6% (95% CI, 2.4–2.8) in women, as estimated by the log‐linear regression model. During the total study period of 21 years, the total stroke incidence decreased by 36% in both men (from 489 to 315 cases per 100 000 population) and women (from 344 to 220 cases per 100 000 population; Figure 1).

Age‐standardized incidence per 100 000 population for each of the stroke subtypes decreased for both men and women during the total study period from 2001 to 2021 (Table 1). The largest relative decrease in the absolute observed incidence per 100 000 population was found for unspecified stroke by 85% in men (70 cases) and 83% (56 cases) in women, followed by ischemic stroke by 27% (94 cases) in men and 28% (66 cases) in women, and intracerebral hemorrhage by 23% (17 cases) in men and 13% (6 cases) in women (Table 1).

**Table 1:** Age‐Standardized Incidence per 100 000 Population in 2001, 2011, and 2021 With Average Annual Percentage Change and Difference in Incidence Between 2001 and 2021 for Total Stroke, Stroke Subtypes, and TIA by Sex

		Age‐standardized incidence per 100 000 population			Average annual relative change (%) with 95% CI†	Difference in the observed age‐standardized incidence per 100 000 between 2001 and 2021 (proportion relative to 2001 [%])‡
		2001	2011	2021		
Total stroke	Men	489.4	407.8	315.4	−2.4 (−2.6 to −2.3)*	−174 (−35.6%)
	Women	344.0	283.2	219.8	−2.6 (−2.8 to −2.4)*	−124 (−36.1%)
Ischemic stroke	Men	351.8	308.4	257.7	−1.8 (−2.0 to −1.7)*	−94 (−26.7%)
	Women	237.9	209.1	172.3	−2.0 (−2.2 to −1.8)*	−66 (−27.6%)
Intracerebral hemorrhage	Men	73.2	68.4	56.3	−1.1 (−1.3 to −0.8)*	−17 (−23.1%)
	Women	49.8	42.9	43.5	−0.7 (−1.1 to −0.4)*	−6 (−12.7%)
Unspecified stroke	Men	81.6	44.5	12.0	−9.9 (−10.7 to −9.0)*	−70 (−85.3%)
	Women	67.1	39.5	11.4	−9.1 (−10.0 to −8.2)*	−56 (−83.1%)
TIA	Men	101.7	143.6	109.7	0.4 (−0.4 to 1.3)	8 (7.9%)
	Women	79.5	117.3	90.9	1.3 (0.3 to 2.3)*	11 (14.3%)

TIA indicates transient ischemic attack.

* *P*<0.05.

^†^ Calculated by log‐linear regression, using information from all years between 2001 and 2021.

^‡^ Calculated as the difference of the observed age‐standardized incidence per 100 000 population in 2021 and 2001.

**Figure 2:**
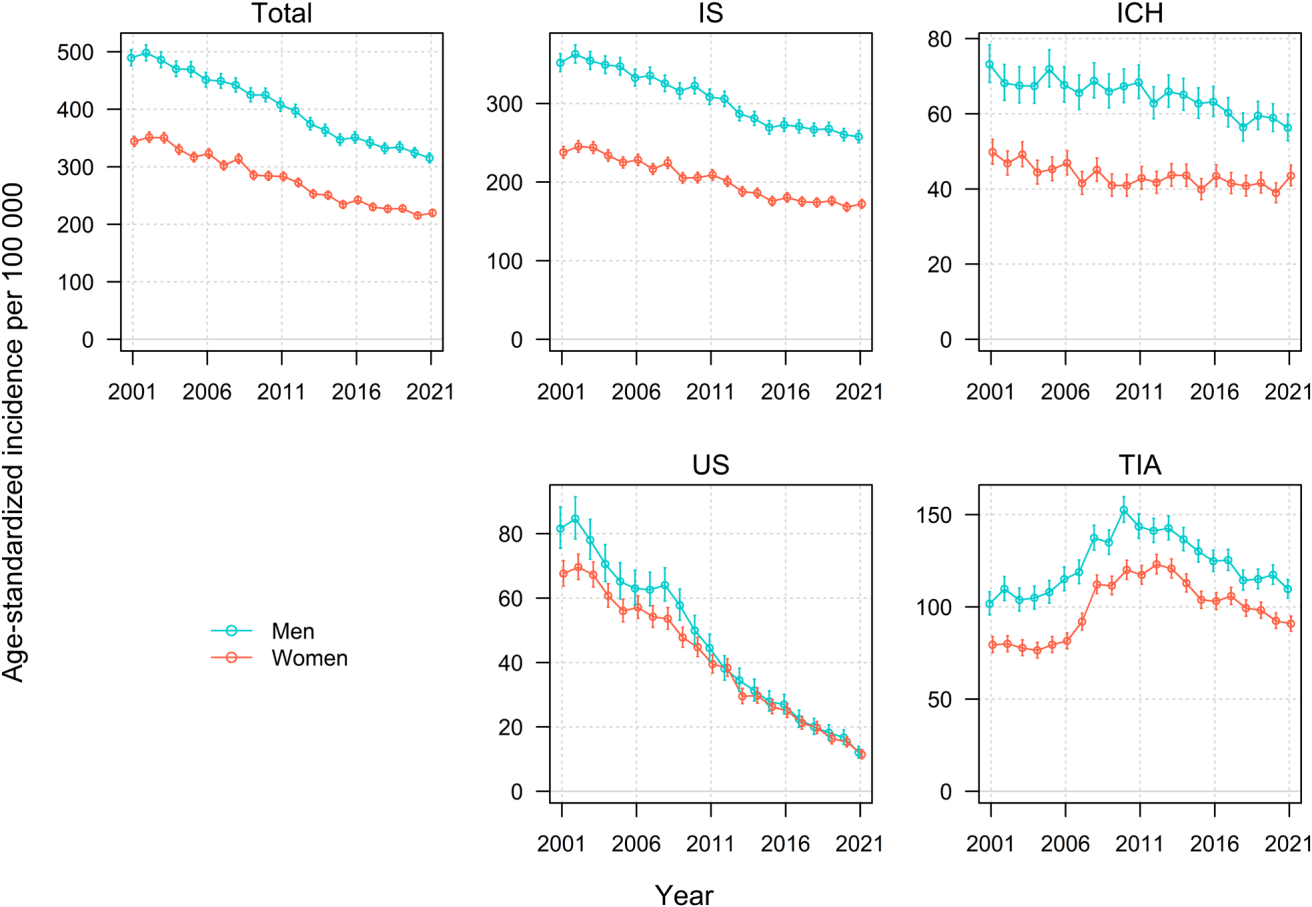
Age‐standardized incidence per 100 000 population (95% CI) stratified by sex for total stroke, ischemic stroke, intracerebral hemorrhage, unspecified stroke, and TIA for women and men in Norway, 2001 to 2021. ICH indicates intracerebral hemorrhage; IS, ischemic stroke; and US, unspecified stroke.

During the total study period, the incidence of TIA increased by 8% in men, and 14% in women, corresponding to an annual increase of 0.4% (−0.4% to 1.3%) and 1.3% (0.3%–2.3%), respectively. However, the graphs were nonlinear, with an inverted U shape, and the increase was evident only from 2001 to 2012 (Figure 2). From 2013 to 2021, the incidence of TIA decreased for both sexes, by 23.1% in men and 24.8% in women (Figure S2).

**Figure 3:**
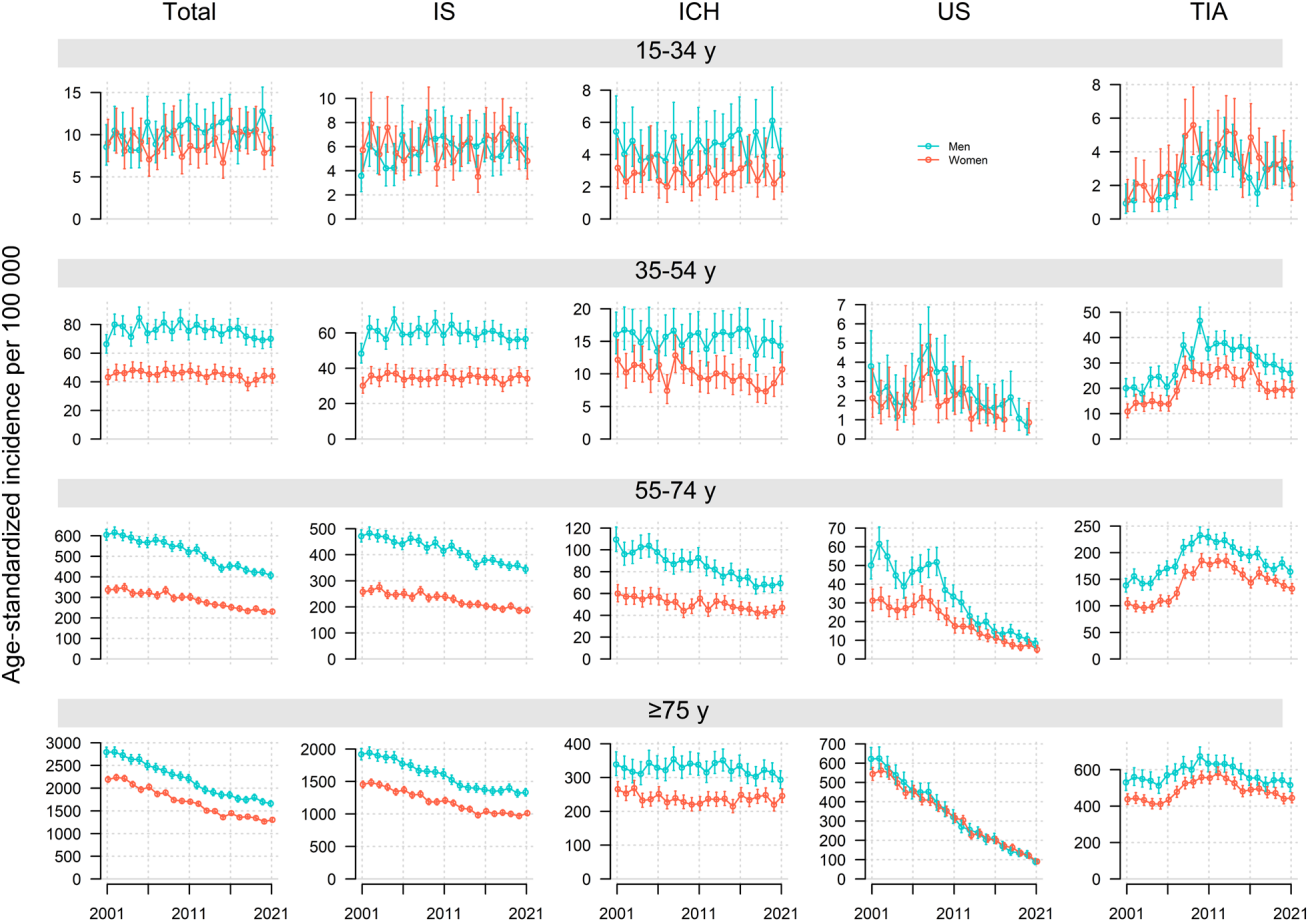
Age‐standardized incidence per 100 000 population (95% CI) for total stroke, stroke subgroups and TIA by sex and age group in Norway, 2001 to 2021. ICH indicates intracerebral hemorrhage; IS, ischemic stroke; TIA, transient ischemic attack; Total, total stroke; and US, unspecified stroke.

In age‐ and sex‐stratified analyses of total stroke, we observed annual decreases in incidence per 100 000 population ranging from 2.1% to 3.0% for individuals aged >55 years in both sexes (Table 2; Figure 3). For men aged 15 to 34 years, we observed an annual increase of 0.9% (0.0%–1.8%), while men aged 35 to 54 years showed a stable trend. Among women, the 15‐ to 34‐year age group exhibited a stable trend, whereas in women aged 35 to 54 years, there was an annual decrease of 0.5% (−0.8% to −0.1%).

**Table 2:** Average Annual Percentage Change (95% CI) in Incidence per 100 000 Population for Total Stroke, Stroke Subtypes, and TIA by Sex and Age Group, 2001 to 2021

Type	Sex	Age 15–34 y	Age 35–54 y	Age 55–74 y	Age ≥75 y
Total stroke	Men	0.9 (0.0 to 1.8)	−0.3 (−0.7 to 0.1)	−2.2 (−2.4 to −2.0)*	−2.8 (−3.0 to −2.6)*
	Women	0.0 (−1.1 to 1.1)	−0.5 (−0.8 to −0.1)*	−2.2 (−2.4 to −2.0)*	−3.0 (−3.2 to −2.7)*
	Both	0.5 (0.0 to 1.0)	−0.4 (−0.7 to 0.0)	−2.1 (−2.3 to −1.9)*	−2.8 (−3.0 to −2.6)*
Ischemic stroke	Men	1.6 (0.3 to 2.8)*	0.0 (−0.5 to 0.5)	−1.7 (−1.9 to −1.5)*	−2.1 (−2.3 to −1.9)*
	Women	−0.3 (−2.0 to 1.5)	0.0 (−0.4 to 0.4)	−1.9 (−2.1 to −1.6)*	−2.2 (−2.5 to −2.0)*
	Both	0.6 (−0.4 to 1.5)	0.0 (−0.4 to 0.4)	−1.7 (−1.9 to −1.5)*	−2.1 (−2.3 to −1.9)*
Intracerebral hemorrhage	Men	0.5 (−0.8 to 1.8)	−0.3 (−0.9 to 0.2)	−2.4 (−2.7 to −2.1)*	−0.4 (−0.7 to −0.1)*
	Women	0.1 (−1.3 to 1.4)	−1.4 (−2.3 to −0.4)*	−1.5 (−1.9 to −1.0)*	−0.3 (−0.7 to 0.1)
	Both	0.5 (−0.4 to 1.4)	−0.7 (−1.3 to −0.2)*	−2.0 (−2.3 to −1.7)*	−0.2 (−0.5 to 0.0)
Unspecified stroke	Men	Few individuals	−9.3 (−12.3 to −6.2)*	−10.1 (−11.2 to −8.9)*	−9.8 (−10.6 to −9.0)*
	Women	Few individuals	−7.7 (−10.0 to −5.2)*	−9.8 (−11.0 to −8.6)*	−9.1 (−9.9 to −8.3)*
	Both	Few individuals	−8.3 (−10.8 to −5.7)*	−9.9 (−10.9 to −8.8)*	−9.2 (−10.0 to −8.4)*
TIA	Men	15.6 (7.3 to 24.5)*	2.3 (0.8 to 3.7)*	0.9 (−0.1 to 1.9)	−0.2 (−0.7 to 0.4)
	Women	4.4 (1.5 to 7.4)*	2.9 (1.3 to 4.6)*	2.2 (1.0 to 3.4)*	0.5 (−0.2 to 1.2)
	Both	7.3 (3.9 to 10.8)*	2.5 (1.0 to 4.0)*	1.5 (0.4 to 2.6)*	0.2 (−0.4 to 0.8)

TIA indicates transient ischemic attack.

* *P*<0.05.

We found notable sex differences in stroke incidence, most pronounced in the age groups 35 to 54 and 55 to 74 years, where the incidence in men was approximately twice the incidence in women. Compared with older age groups, unspecified stroke was rare in younger age groups, while intracerebral hemorrhage contributed to a larger proportion of strokes in these groups. There was no clear sex difference in incidence in the youngest age group, 15 to 34 years (Figure 3; Table S1).

### Age‐ and Sex‐Stratified Analyses of Stroke Subtypes and TIA

Incidence per 100 000 population of ischemic stroke decreased by 1.7% to 2.3% per year in the age groups ≥55 years in both sexes. For those aged <55 years, the incidence was stable over time in women and for men aged 35 to 54 but increased for men aged 15 to 34 years by 1.6% (0.3–2.8) annually.

Incidence of intracerebral hemorrhage decreased for women in the 35‐ to 54‐year age group, for both sexes in the 55‐ to 74‐year age group, and for the oldest men. In contrast, the incidence remained stable for the youngest and oldest women, as well as for younger men aged <55 years.

In subgroup analyses of the oldest population (75–84 and ≥85 years), the incidence of hemorrhagic stroke declined for both sexes in the 75‐ to 84‐year age group, but remained stable in the ≥85 year age group (Figure S1).

For TIA, the trends in incidence were nonlinear across all age groups, with an inverted U shape with an increase up to 2012 followed by a decrease (Figure S2). Overall, the change in incidence was nonsignificant in the oldest strata of the population, while significant increases followed by decreases were observed for both men and women aged 15 to 34 and 35 to 54 years, as well as for women aged 55 to 74 years (Table 2; Figure 3).

### Other Analyses

The total stroke population had a lower educational level compared with the general population. Over time, the educational level increased in both populations (Figure S3).

During the study period, the proportion with incident out‐of‐hospital stroke deaths decreased from 10.5% to 4.3%. Among cases of incident out‐of‐hospital stroke deaths, the majority were classified as unspecified strokes (81.6%), followed by intracerebral hemorrhage (9.4%) and ischemic stroke (9.0%).

Among hospitalized patients with incident stroke, the proportion with ischemic stroke increased from 78.2% in 2001 to 82.4% in 2021, as did the proportion of hemorrhagic strokes (13.9%–16.0%). Meanwhile there was a substantial decrease in the proportion registered with unspecified stroke (7.9%–1.6%).

We used a fixed lookback period of 7 years to define incident stroke. A total of 7.7% of those with an incident stroke in 2021 actually had a stroke before the lookback period, back to 1994. However, this “false‐positives error” appeared to be stable during the study period (Figure S5).

## Discussion

From 2001 to 2021, we observed a significant decrease in the incidence of total stroke in the Norwegian population ≥15 years. The decrease was primarily driven by a reduction in ischemic stroke incidence, particularly among individuals aged ≥55 years. In addition, there was a significant overall decrease in incidence for all stroke subtypes (ischemic, intracerebral hemorrhage, and unspecified stroke) in both men and women. However, a concerning increase in ischemic stroke was observed in young men aged 15 to 34 years. Furthermore, we found overall less favorable time trends in younger age groups compared with older age groups. For TIA, both sexes and all age groups experienced a sharp rise in incidence before 2012, followed by a substantial decrease from 2013 onward.

With regard to the increase in ischemic stroke incidence among men in the youngest age group, we also found a significant increase in TIA in the same age group. This means that the increase in stroke is less likely to be explained by a shift in diagnosis from TIA to stroke. Although one should keep in mind the possibility of false positives due to multiple testing, the result is in line with several other studies. In the Tromsø Study, the incidence of ischemic stroke among individuals aged 30 to 49 years increased for women and showed a trend toward an increase in men from 1977 to 2010.^23^ Stable or declining overall trends of annual number of prevalent cases hospitalized with stroke or TIA in the Norwegian population aged <55 years from 2010 to 2015 were reported in national register‐based analyses by Barra et al,^25^ but this study covered a short time horizon of only 6 years. In a Swedish register study, incidence of ischemic stroke (using 7‐year fixed lookback period) decreased in the individuals aged ≥65 years, but the decline was less steep for individuals aged 45 to 64 years, whereas the incidence increased for the individuals aged 18 to 44 years.^6^ A study using data from the Danish Stroke Registry found stable incidence of ischemic stroke and intracerebral hemorrhage during 2005 to 2018 in adults aged <50 years, while it decreased in adults ≥70 years of age.^7^ Yet another Danish nationwide study among individuals aged 15 to 30 years reported increased incidence of ischemic stroke and TIA, and stable incidence of intracerebral hemorrhage during 1994 to 2012.^8^ In line with these studies, a recent systematic review concluded that temporal trends in stroke incidence are diverging by age in high‐income countries, with less favorable trends at younger versus older ages.^26^

One possible explanation for the trend toward increased ischemic stroke in young individuals could be improved detection of minor strokes in younger individuals, aided by the widespread availability of brain imaging, particularly diffusion‐weighted magnetic resonance imaging (MRI). This notion is supported by studies reporting that a significant proportion of strokes in younger age groups are indeed minor events.^7^, ^24^ However, then one would expect a decrease also in younger ages during the recent years of stable MRI availability.

Risk factors for ischemic stroke in younger adults are not unique and overlap with those of older adults.^30^ A recent study found that vascular risk factors were highly prevalent in younger patients with stroke (aged 18–55 years), in both patients with undetermined and determined stroke pathogenesis.^31^ Interestingly, the proportion of TIAs and strokes among those aged <55 years without known vascular risk factors increased significantly over time in a recent study from England,^24^ and other studies have also shown that most ischemic strokes in the young are of unknown cause.^32^, ^33^ However, some risk factors are more common in younger adults compared with older adults. Psychostimulant use was associated with nearly a fifth of cases of fatal strokes in young adults (aged 15–44 years) in a study from Australia.^34^ In accordance with this, a study from the United States found a strong association between acute cocaine use and risk of stroke.^35^ Other suggested explanations for the increase of stroke in the young are reduced physical activity, increasing obesity, excess alcohol consumption, and smoking.^30^, ^36^ Common risk factors for all stroke subtypes include higher age, hypertension, abdominal obesity, current smoking, and high alcohol intake.^37^ In the global INTERSTROKE (Risk Factors for Ischemic and Intracerebral Hemorrhagic Stroke in 22 Countries) study, hypertension was a stronger risk factor for intracerebral hemorrhages, as opposed to ischemic strokes where current smoking, diabetes, unfavorable blood lipids, and cardiac causes such as atrial fibrillation (AF) were stronger risk factors.^37^ Changing trends in stroke risk factors such as a favorable decrease in the proportion of smokers and decreasing levels of systolic blood pressure, but also unfavorable increasing proportions with diabetes and higher level of body mass index, explained 57% of the decline in ischemic stroke incidence during 1995 to 2012 in the regional population‐based Norwegian Tromsø Study.^38^ A steeper decline in blood pressure level in women than men in the Tromsø Study may have contributed to the finding of declining incidence of intracerebral hemorrhages in younger women only.^39^

AF and stroke share the same risk factors, except that AF is a risk factor for stroke. Our research group found that trends in incidence of AF in Norway were stable during 2004 to 2014 for all ages. However, a worrying increase was observed in men aged <45 years.^40^ This is the same sex and age group that demonstrates an increase in ischemic stroke incidence in our study. Furthermore, in stratified analyses for the period 2001 to 2014, we found decreasing incidence in non‐AF related ischemic stroke and intracerebral hemorrhage but stable incidence for AF‐related strokes.^41^ Our research group has previously documented increased use of oral anticoagulant therapy in patients with AF in Norway during 2010 to 2015 and 2012 to 2017.^11^, ^12^ However, incidence of intracranial hemorrhage in patients with AF have remained stable, in line with our results.^11^

We found a significant overall increase in incidence of TIA for all ages for the total time period; however, during 2012 to 2021, the incidence was decreasing for all ages. A study on young adults from Denmark found an increase in TIA incidence rate until 2012; however, updated incidence rates are lacking.^8^ The previously mentioned Norwegian nationwide register study by Rand et al found decreasing annual number of age‐standardized prevalent cases with TIA hospitalization during 2010 to 2015.^20^ A possible explanation for the observed increase until 2012 might be improved stroke awareness and changes in clinical practice, so that TIA and less severe strokes were increasingly hospitalized during that period.^25^ From 2013 onward, the decline in incidence of TIA is in line with or even more pronounced than the decline in incident ischemic strokes in our data, suggesting that improvements in the use of diagnostic brain imaging may have influenced these trends.^26^ In addition, in 2010, the first national stroke guideline was published in Norway and suggested that some patients with TIA, with low risk of stroke (ABCD [age, blood pressure, clinical features, duration of symptoms, diabetes] score, 0–3) could be handled as an outpatient.^42^ However, only a few hospitals have handled patients with TIA as outpatients to our knowledge, and the number of patients has been quite low and will probably not explain the decline.

With regard to the subtypes of stroke, a Norwegian study of prevalent stroke cases in hospitalized individuals suggested decreasing age‐standardized total stroke driven by a decline in ischemic stroke during 2010 to 2015. Intracerebral hemorrhages remained stable.^20^ This study did not use a lookback period; thus, the prevalent cases were a mixture of first and recurrent events. In a population‐based regional health examination study for the northern part of Norway, the Tromsø Study, a decline in ischemic stroke incidence from 1995 to 2013 was also reported, while incidence of intracerebral hemorrhage declined in women and was stable in men.^23^, ^39^, ^43^

Total stroke incidence includes both unspecified stroke and specified stroke subtypes, and temporal trends in total stroke are thus not influenced by potential shifts from unspecified to specified stroke subtypes. Temporal changes in incidence of unspecified stroke must be accounted for when interpreting time trends in incident ischemic strokes and intracerebral hemorrhages. Diagnostic imaging has become more available,^16^ and we may expect a reclassification of cases previously classified as unspecified stroke into ischemic stroke or cerebral hemorrhage, resulting in an increase in the number of incident stroke cases with ischemic strokes or intracerebral hemorrhage. A major part of unspecified strokes are incident cases presenting as death from stroke without a prior stroke hospitalization. The decrease in overall out‐of‐hospital strokes may be linked to improved prehospital care and availability of time‐critical treatments,^44^ allowing more patients with stroke to reach the hospital alive.

We found decreasing mean and median age in incident stroke cases from 2001 to 2021. This may be due to increased availability and use of diagnostic imaging with earlier detection of strokes, at least in the younger patients. It may also be that imaging in fact reduces the diagnosis of stroke in the oldest patients with high comorbidity and unclear clinical presentation.

Major strengths of our study include use of nationwide health registry data with a long observation period, which allows for an adequate identification of incident events by applying a reasonably long fixed lookback period.^45^ We included hospitalization with stroke as the primary or secondary diagnosis and also captured strokes that were accompanied with other main conditions. In addition, we included out‐of‐hospital deaths due to stroke. This may have provided us with a more complete number of stroke cases. Also, we excluded stroke as a secondary diagnosis when the primary diagnosis was previous stroke or rehabilitation, to ensure correctness.^46^ However, some limitations must be considered. We do not have detailed information on race and ethnicity beyond Western/non‐Western migration background, which affects the generalizability to populations that are not predominantly racially and ethnically homogeneous. Furthermore, temporal changes in clinical guidelines, treatment strategies, access to diagnostic imaging, coding practice, and treatment of patients in primary or secondary care need to be taken into account when interpreting register data from patient administrative systems. Although our data did not include imaging confirmation of strokes, a recent study has shown high sensitivity (86.4%) and positive predictive value (84.2%) for the Norwegian Patient Registry, compared with a gold standard of cases ascertained through expert review of medical records, supporting the validity of such administrative data for stroke diagnoses.^47^

## Conclusions

Total stroke incidence per 100 000 population declined from 2001 to 2021 in the Norwegian adult population. However, less favorable trends were observed in younger age groups compared with older age groups, particularly concerning an increase in ischemic stroke among men aged 15 to 34 years. These trends raise important questions and warrant further investigation.

## Sources of Funding

This research has received a public grant from the Research Council of Norway, grant number 336283.

## Disclosures

None.

## Supporting information

Table S1
Figures S1–S5
Supplemental Methods and Results
