# Supplementary material for: Nationwide Trends in Incidence of Stroke and Transient Ischemic Attack in Younger and Older Adults in Norway 2001 to 2021
Source: J Am Heart Assoc. 2025 Dec 11;14(24):e041029. doi: 10.1161/JAHA.125.041029 (PMC12826924; doi:10.1161/JAHA.125.041029)
Supplement: Supplementary file 1 — Table S1 Figures S1–S5 Supplemental Methods and Results [file JAH3-14-e041029-s001.pdf]

# Supplemental Material

**Supplemental methods**

Statistics

Annual relative change in incidence was estimated by weighted log-linear regression as follows: Let $I_Y$ denote the age-standardized incidence in year Y, Y=2001,…,2021, and var($I_Y$) the corresponding variance estimate provided by the ageadjust.direct function. The weighted regression equation is then

$$\log(I_Y) = \alpha + \beta Y + \epsilon_Y, \quad (1)$$

where $\beta$ represents the annual increase in $\log(I_Y)$, and $\epsilon_Y$ is a normally distributed random error with mean 0 and variance $\sigma^2 w_Y$, where $w_Y = 1/\text{var}(I_Y)$ is the weight. The annual percentage change in incidence is then $\delta = (\exp(\beta)-1)*100$. The estimate of $\beta$ was obtained in R as `coef(obj)[2]`, where

```
obj <- lm(logIY ~ Y, weight=invVarIY)
```

where invVarIY = 1/var($I_Y$).

As a sensitivity analysis we estimated 95% confidence intervals for the annual percentage change $\delta$ by taking the first order autocorrelation in the residuals $\hat{\epsilon}_Y$ in (1) into account. This was done using the `coefci` function of the `lmtest` package in R to obtain the autocorrelation consistent confidence interval CI for $\beta$ in (1) as

```
CI <- coefci(obj, vcov=NeweyWest, lag=1)
```

The results are presented in Supplemental figure S4.

Definition of demographic variables

Demographic variables (income, education and country background) by sex and age groups for the first six and the last six years of the period for total stroke are presented in Supplemental figure S3.

The age distribution of the stroke population was skewed within each 20-year age group, with an overweight of older individuals. To compare the proportions of the stroke population relative to the population at risk (general population), the general population was down-sampled by excluding randomly selected younger individuals so that the proportion of the population that were in each one-year age group was similar in the general population as in the general population in each calendar year.

Within each age group, the proportion with e.g. low education in the first six-year period was calculated as the number of stroke cases with low education divided by the total number of stroke cases with information on education in that age group. Correspondingly we calculated the proportion with low education among the age-weighted general population sample.

The demographic variables were defined the following way;

*Education* (the calendar year before stroke year, i.e. 2000-2005 and 2015-2020):

- Compulsory: Lower secondary education, 12th class level, or shorter.
- Intermediate: Upper secondary, 11th class level to post-secondary not higher education 14th class level +
- High: At least first stage of higher education, undergraduate level, 14th class level +

https://www.ssb.no/en/utdanning/artikler-og-publikasjoner/norwegian-standard-classification-of-education-2016

*Personal income after taxes* (the calendar year before stroke year, i.e. 2000-2005 and 2015-2020):

- Low (<2G)
- Medium (2G – <4G)
- High (≥ 4G)

Where G is the basic amount in the National Insurance scheme, which in 2000, 2010 and 2020 were (NOK) 48 377, 74 721 and 100 853, respectively. For all years, see

https://www.skatteetaten.no/en/rates/national-insurance-scheme-basic-amount/

*Region of origin*: Country of origin, grouped as

- Norway: Country of origin is Norway.
- G1 (Western): Country/region of origin is another Nordic country, EU/EFTA, UK, USA, Canada, Australia, or New Zealand
- G2 (Non-Western): Region of origin is Europe excluding EU/EFTA and the UK, Africa, Asia, America excluding the USA and Canada, Oceania excluding Australia and NZ, polar regions

where country of origin is defined as follows:

- For persons born abroad: their own country of birth.
- For persons born in Norway with at least one parent born abroad: the country of birth of their parents (mother if the parents have different countries of birth).
- For persons born in Norway with no parents born abroad: the country of birth of the first foreign-born person found in the order grandmother, grandfather, great-grandmother or great-grandfather.
- For persons born in Norway with no parents/grandparents/great-grandparents born abroad: Norway.

**Supplemental results**

Supplemental figure S3 shows educational inequalities, with lower education in the stroke population than in the general population for both sexes. This applies for all both men and women aged 15 years and older, and all age groups. The proportion with low education decreased over time both in the stroke population and in the general population for both men and women aged 15 years and older, and for the age groups above 35 years. However, for the youngest age groups the proportion with low education in the stroke population in the first time period (2001-2006) was similar to the proportion in the last time period (2016-2021) for both men and women. In general, the educational level in the population increased over time.

Regarding income, we found a higher proportion with high income in the general population than in the stroke population for both men and women aged 15 years and older. In the youngest and the oldest age groups there were no major differences for men nor women between the stroke population and the general population. The proportion with high income increased over time in both the stroke population and the general population.

Regarding immigration background, we found a smaller proportion with Norwegian background in the stroke population and in the general population in the first time period and correspondingly larger proportions of persons with Western and Non-Western background in the last time period, consistent with increased immigration. There were no major differences in the proportion with Western and Non-Western background between the stroke population and the general population.

Supplemental figure S4 shows the sensitivity analysis where we estimated 95% confidence intervals for the annual percentage change by taking the autocorrelation in the residuals between adjacent years into account. The results are very similar to the main analysis, however, total stroke for the youngest age group increases significantly in the sensitivity analysis, while it was only borderline significant in the main analysis. The increase of ischemic stroke for men in the youngest age group remains significant. For TIA the increase over the total period is not significant for men and women 35-54 years in the sensitivity analysis. For ICH the decrease for women 35-54 and men 75+ are no longer significant in the sensitivity analysis. However, the trends are similar to the main analysis.

Table S1. Age-standardized incidence per 100,000 population with 95% CI for total stroke (Total), ischemic stroke (IS), intracerebral hemorrhage (ICH), unspecified stroke (US) and transient ischemic attack (TIA) by sex and age group in Norway, 2001 to 2021.

Years and subgroups with less than five individuals in each group are not shown.

| sex | age | year | Total | IS | ICH | US | TIA |
|---|---|---|---|---|---|---|---|
| Men | 15-34 | 2001 | 8.54 (6.39-11.2) | 3.58 (2.27-5.41) | 5.43 (3.73-7.65) | | 0.93 (0.34-2.08) |
| Men | 15-34 | 2002 | 10.5 (8.10-13.4) | 6.14 (4.36-8.43) | 4.03 (2.60-5.98) | | 1.10 (0.44-2.31) |
| Men | 15-34 | 2003 | 9.82 (7.51-12.6) | 5.45 (3.77-7.64) | 4.85 (3.27-6.95) | | |
| Men | 15-34 | 2004 | 8.16 (6.06-10.8) | 4.21 (2.75-6.20) | 3.64 (2.28-5.53) | | |
| Men | 15-34 | 2005 | 8.18 (6.07-10.8) | 4.25 (2.77-6.25) | 3.79 (2.40-5.72) | | 1.15 (0.46-2.40) |
| Men | 15-34 | 2006 | 11.5 (8.92-14.5) | 6.96 (5.01-9.43) | 4.01 (2.57-5.99) | | 1.32 (0.57-2.62) |
| Men | 15-34 | 2007 | 8.87 (6.66-11.6) | 5.27 (3.60-7.46) | 3.60 (2.25-5.48) | | 1.46 (0.67-2.80) |
| Men | 15-34 | 2008 | 10.8 (8.30-13.7) | 5.35 (3.66-7.56) | 5.10 (3.46-7.25) | | 3.17 (1.91-4.96) |
| Men | 15-34 | 2009 | 9.93 (7.59-12.8) | 6.65 (4.77-9.03) | 3.45 (2.13-5.27) | | 2.16 (1.15-3.69) |
| Men | 15-34 | 2010 | 11.1 (8.65-14.1) | 6.65 (4.77-9.03) | 4.15 (2.71-6.09) | | 3.64 (2.31-5.48) |
| Men | 15-34 | 2011 | 11.8 (9.26-14.8) | 6.88 (4.98-9.28) | 4.89 (3.34-6.93) | | 3.95 (2.55-5.85) |
| Men | 15-34 | 2012 | 10.8 (8.44-13.7) | 6.29 (4.51-8.55) | 4.19 (2.79-6.08) | | 2.90 (1.74-4.54) |
| Men | 15-34 | 2013 | 10.3 (7.96-13.0) | 5.66 (3.98-7.81) | 4.75 (3.25-6.72) | | 4.18 (2.78-6.05) |
| Men | 15-34 | 2014 | 11.0 (8.66-13.8) | 6.40 (4.63-8.63) | 4.61 (3.15-6.52) | | 3.84 (2.51-5.64) |
| Men | 15-34 | 2015 | 11.5 (9.07-14.3) | 6.02 (4.32-8.17) | 5.15 (3.60-7.14) | | 3.06 (1.89-4.69) |
| Men | 15-34 | 2016 | 11.9 (9.48-14.8) | 6.52 (4.75-8.73) | 5.54 (3.94-7.59) | | 2.45 (1.43-3.93) |
| Men | 15-34 | 2017 | 8.57 (6.55-11.0) | 5.10 (3.57-7.08) | 3.46 (2.24-5.13) | | 1.54 (0.77-2.77) |
| Men | 15-34 | 2018 | 10.6 (8.36-13.3) | 5.20 (3.66-7.18) | 5.41 (3.85-7.41) | | 2.99 (1.85-4.58) |
| Men | 15-34 | 2019 | 10.4 (8.18-13.1) | 6.37 (4.64-8.53) | 3.91 (2.60-5.66) | | 3.27 (2.07-4.92) |
| Men | 15-34 | 2020 | 12.8 (10.3-15.7) | 6.65 (4.90-8.82) | 6.10 (4.43-8.20) | | 2.96 (1.83-4.52) |
| Men | 15-34 | 2021 | 9.71 (7.57-12.3) | 5.82 (4.20-7.88) | 3.89 (2.58-5.63) | | 3.07 (1.93-4.66) |
| Women | 15-34 | 2001 | 9.05 (6.81-11.8) | 5.72 (3.98-7.99) | 3.17 (1.91-4.98) | | 1.12 (0.45-2.36) |
| Women | 15-34 | 2002 | 10.2 (7.83-13.1) | 7.90 (5.82-10.5) | 2.32 (1.27-3.93) | | 2.10 (1.12-3.64) |
| Women | 15-34 | 2003 | 8.07 (5.95-10.7) | 5.37 (3.67-7.61) | 2.87 (1.67-4.62) | | 1.98 (1.02-3.49) |
| Women | 15-34 | 2004 | 10.2 (7.85-13.2) | 7.58 (5.54-10.1) | 2.83 (1.65-4.56) | | 1.12 (0.45-2.35) |
| Women | 15-34 | 2005 | 9.15 (6.89-11.9) | 5.47 (3.76-7.71) | 3.84 (2.43-5.79) | | 2.51 (1.40-4.17) |
| Women | 15-34 | 2006 | 7.07 (5.09-9.58) | 4.84 (3.24-6.98) | 2.38 (1.30-4.02) | | 2.69 (1.54-4.39) |
| Women | 15-34 | 2007 | 7.98 (5.86-10.6) | 5.79 (4.00-8.11) | 2.01 (1.04-3.54) | | 2.23 (1.19-3.83) |
| Women | 15-34 | 2008 | 9.54 (7.20-12.4) | 5.59 (3.84-7.86) | 3.08 (1.83-4.89) | | 4.95 (3.31-7.12) |
| Women | 15-34 | 2009 | 10.4 (8.01-13.4) | 8.28 (6.12-10.9) | 2.86 (1.66-4.58) | | 5.59 (3.85-7.86) |
| Women | 15-34 | 2010 | 7.38 (5.36-9.92) | 4.23 (2.74-6.25) | 2.13 (1.13-3.66) | | 3.33 (2.03-5.16) |
| Women | 15-34 | 2011 | 8.67 (6.49-11.3) | 6.07 (4.27-8.39) | 2.59 (1.48-4.23) | | 2.95 (1.75-4.68) |
| Women | 15-34 | 2012 | 8.15 (6.06-10.7) | 4.79 (3.23-6.85) | 3.19 (1.95-4.94) | | 4.43 (2.92-6.46) |
| Women | 15-34 | 2013 | 8.66 (6.52-11.3) | 6.14 (4.36-8.41) | 2.22 (1.21-3.74) | | 5.22 (3.59-7.34) |
| Women | 15-34 | 2014 | 9.58 (7.34-12.3) | 6.68 (4.83-9.01) | 2.75 (1.63-4.36) | | 5.10 (3.51-7.17) |
| Women | 15-34 | 2015 | 6.67 (4.84-8.96) | 3.51 (2.22-5.28) | 2.85 (1.71-4.47) | | 2.32 (1.30-3.84) |
| Women | 15-34 | 2016 | 10.3 (8.04-13.1) | 6.92 (5.06-9.24) | 3.14 (1.94-4.82) | | 4.85 (3.32-6.86) |
| Women | 15-34 | 2017 | 10.4 (8.07-13.1) | 6.56 (4.76-8.82) | 3.50 (2.24-5.23) | | 3.65 (2.36-5.40) |
| Women | 15-34 | 2018 | 9.98 (7.73-12.7) | 7.57 (5.63-9.96) | 2.39 (1.36-3.89) | | 2.94 (1.79-4.56) |
| Women | 15-34 | 2019 | 10.6 (8.26-13.3) | 6.95 (5.11-9.25) | 3.32 (2.08-5.02) | | 3.25 (2.04-4.93) |
| Women | 15-34 | 2020 | 7.85 (5.89-10.2) | 5.80 (4.14-7.90) | 2.20 (1.23-3.63) | | 3.53 (2.26-5.26) |
| Women | 15-34 | 2021 | 8.34 (6.32-10.8) | 4.81 (3.31-6.77) | 2.81 (1.69-4.39) | | 2.04 (1.12-3.44) |

| sex | age | year | Total | IS | ICH | US | TIA |
|---|---|---|---|---|---|---|---|
| Men | 35-54 | 2001 | 66.3 (60.1-72.9) | 48.3 (43.0-54.0) | 16.0 (13.1-19.5) | 3.79 (2.43-5.64) | 20.1 (16.7-23.9) |
| | | 2002 | 80.0 (73.1-87.3) | 63.0 (56.9-69.5) | 16.8 (13.7-20.3) | 2.39 (1.34-3.95) | 20.3 (17.0-24.2) |
| | | 2003 | 78.8 (72.0-86.1) | 61.1 (55.2-67.6) | 16.4 (13.4-19.9) | 2.72 (1.58-4.36) | 17.9 (14.8-21.6) |
| | | 2004 | 71.3 (64.9-78.2) | 56.5 (50.8-62.7) | 14.9 (12.0-18.2) | 1.90 (0.98-3.34) | 24.1 (20.5-28.3) |
| | | 2005 | 84.8 (77.7-92.3) | 67.9 (61.6-74.6) | 16.8 (13.7-20.2) | 1.76 (0.88-3.16) | 24.6 (20.9-28.8) |
| | | 2006 | 73.9 (67.4-80.9) | 59.2 (53.3-65.5) | 13.4 (10.8-16.6) | 2.81 (1.66-4.46) | 20.6 (17.2-24.4) |
| | | 2007 | 76.3 (69.7-83.3) | 59.0 (53.3-65.2) | 15.7 (12.8-19.0) | 4.10 (2.70-5.98) | 25.2 (21.5-29.4) |
| | | 2008 | 81.4 (74.6-88.6) | 62.8 (56.9-69.2) | 16.6 (13.7-20.0) | 4.87 (3.33-6.88) | 37.0 (32.5-41.9) |
| | | 2009 | 75.3 (68.8-82.2) | 59.1 (53.4-65.2) | 14.4 (11.7-17.6) | 3.49 (2.21-5.24) | 31.7 (27.6-36.3) |
| | | 2010 | 83.2 (76.5-90.4) | 66.2 (60.2-72.6) | 15.9 (13.1-19.2) | 3.66 (2.36-5.41) | 46.6 (41.6-52.1) |
| | | 2011 | 75.6 (69.2-82.4) | 58.7 (53.1-64.8) | 16.3 (13.4-19.6) | 2.50 (1.46-4.02) | 35.5 (31.2-40.3) |
| | | 2012 | 79.9 (73.4-86.8) | 64.7 (58.9-71.0) | 13.8 (11.2-16.9) | 2.34 (1.36-3.77) | 37.7 (33.2-42.5) |
| | | 2013 | 75.9 (69.6-82.7) | 59.5 (53.9-65.4) | 16.0 (13.2-19.3) | 2.57 (1.52-4.08) | 37.9 (33.4-42.7) |
| | | 2014 | 77.3 (71.0-84.0) | 60.7 (55.1-66.7) | 16.4 (13.6-19.7) | 1.97 (1.08-3.32) | 35.3 (31.0-39.9) |
| | | 2015 | 73.2 (67.1-79.8) | 57.2 (51.9-63.0) | 15.9 (13.2-19.1) | 1.62 (0.84-2.85) | 36.3 (32.1-41.0) |
| | | 2016 | 76.9 (70.6-83.5) | 60.6 (55.0-66.5) | 16.9 (14.1-20.2) | 1.63 (0.84-2.86) | 35.3 (31.1-39.9) |
| | | 2017 | 77.6 (71.4-84.2) | 61.1 (55.6-67.0) | 16.8 (13.9-20.0) | 1.78 (0.95-3.06) | 32.5 (28.5-37.0) |
| | | 2018 | 71.6 (65.7-78.0) | 59.0 (53.6-64.8) | 12.9 (10.5-15.8) | 2.17 (1.24-3.53) | 29.3 (25.5-33.5) |
| | | 2019 | 70.3 (64.5-76.6) | 55.8 (50.5-61.4) | 15.3 (12.6-18.4) | 1.07 (0.46-2.11) | 29.4 (25.7-33.6) |
| | | 2020 | 69.0 (63.2-75.2) | 56.4 (51.1-62.0) | 15.1 (12.4-18.1) | 0.67 (0.22-1.58) | 27.3 (23.7-31.3) |
| | | 2021 | 70.0 (64.2-76.3) | 56.5 (51.3-62.2) | 14.3 (11.7-17.3) | | 25.9 (22.4-29.9) |
| Women | | 2001 | 43.1 (38.0-48.6) | 30.1 (25.9-34.8) | 12.1 (9.53-15.2) | 2.13 (1.14-3.65) | 10.9 (8.40-13.8) |
| | | 2002 | 46.5 (41.2-52.2) | 35.9 (31.3-41.0) | 10.2 (7.84-13.1) | 1.66 (0.79-3.06) | 14.2 (11.4-17.6) |
| | | 2003 | 46.0 (40.8-51.7) | 34.2 (29.7-39.2) | 11.4 (8.86-14.4) | 2.18 (1.16-3.74) | 13.7 (10.9-17.0) |
| | | 2004 | 48.1 (42.7-53.9) | 37.4 (32.7-42.6) | 11.2 (8.75-14.2) | 1.18 (0.47-2.43) | 14.9 (12.0-18.3) |
| | | 2005 | 47.6 (42.3-53.4) | 37.0 (32.3-42.1) | 9.44 (7.16-12.2) | 2.27 (1.24-3.82) | 13.9 (11.1-17.2) |
| | | 2006 | 45.2 (40.1-50.9) | 33.5 (29.1-38.4) | 11.3 (8.86-14.3) | 1.62 (0.78-2.99) | 13.8 (11.0-17.0) |
| | | 2007 | 44.9 (39.8-50.4) | 35.0 (30.6-40.0) | 7.42 (5.45-9.88) | 3.16 (1.93-4.89) | 19.1 (15.8-22.8) |
| | | 2008 | 48.4 (43.2-54.2) | 33.8 (29.4-38.6) | 12.8 (10.2-16.0) | 3.63 (2.30-5.45) | 28.2 (24.2-32.6) |
| | | 2009 | 45.8 (40.7-51.3) | 34.0 (29.6-38.8) | 11.0 (8.58-13.9) | 1.71 (0.86-3.08) | 26.6 (22.8-31.0) |
| | | 2010 | 46.4 (41.3-52.0) | 34.7 (30.4-39.6) | 10.6 (8.25-13.4) | 1.99 (1.06-3.42) | 25.7 (22.0-29.9) |
| | | 2011 | 47.5 (42.4-53.0) | 37.1 (32.6-42.1) | 9.40 (7.22-12.1) | 2.30 (1.29-3.81) | 25.2 (21.5-29.4) |
| | | 2012 | 45.5 (40.5-50.9) | 34.5 (30.2-39.3) | 9.19 (7.02-11.8) | 2.71 (1.61-4.30) | 27.6 (23.8-32.0) |
| | | 2013 | 43.2 (38.4-48.5) | 33.6 (29.4-38.4) | 10.1 (7.83-12.8) | 1.05 (0.42-2.18) | 28.4 (24.5-32.8) |
| | | 2014 | 46.8 (41.8-52.3) | 36.1 (31.7-40.9) | 10.0 (7.76-12.7) | 1.59 (0.79-2.87) | 24.3 (20.7-28.3) |
| | | 2015 | 45.3 (40.4-50.6) | 35.3 (31.0-40.1) | 8.93 (6.83-11.5) | 1.44 (0.69-2.67) | 23.9 (20.3-27.9) |
| | | 2016 | 44.5 (39.7-49.8) | 34.7 (30.4-39.3) | 9.67 (7.49-12.3) | 1.17 (0.50-2.31) | 29.5 (25.6-33.9) |
| | | 2017 | 44.2 (39.4-49.4) | 34.8 (30.6-39.5) | 8.91 (6.83-11.4) | 1.01 (0.40-2.09) | 22.2 (18.9-26.0) |
| | | 2018 | 38.3 (33.9-43.2) | 30.9 (26.9-35.3) | 7.55 (5.65-9.88) | | 18.8 (15.7-22.3) |
| | | 2019 | 41.3 (36.7-46.3) | 34.4 (30.2-39.0) | 7.31 (5.46-9.59) | | 19.3 (16.2-22.8) |
| | | 2020 | 44.2 (39.4-49.4) | 36.1 (31.8-40.8) | 8.56 (6.55-11.0) | 0.86 (0.32-1.88) | 19.8 (16.6-23.3) |
| | | 2021 | 43.9 (39.1-49.0) | 34.1 (30.0-38.7) | 10.7 (8.41-13.4) | | 19.3 (16.2-22.8) |

| sex | age | year | Total | IS | ICH | US | TIA |
|---|---|---|---|---|---|---|---|
| Men | 55-74 | 2001 | 605 (579-632) | 472 (449-495) | 109 (98.7-121) | 50.0 (42.8-58.2) | 138 (126-151) |
| | | 2002 | 616 (590-643) | 483 (460-507) | 96.0 (86.0-107) | 61.5 (53.4-70.6) | 156 (143-169) |
| | | 2003 | 602 (576-628) | 472 (450-496) | 97.4 (87.5-108) | 54.8 (47.2-63.3) | 142 (130-155) |
| | | 2004 | 591 (566-618) | 469 (446-492) | 103 (92.3-114) | 44.6 (37.8-52.4) | 142 (130-156) |
| | | 2005 | 571 (546-596) | 449 (427-471) | 104 (93.4-115) | 38.8 (32.5-46.1) | 163 (150-176) |
| | | 2006 | 567 (542-592) | 441 (420-464) | 97.8 (88.1-108) | 46.6 (39.7-54.4) | 170 (157-184) |
| | | 2007 | 581 (556-606) | 463 (441-485) | 90.6 (81.3-101) | 47.9 (41.0-55.6) | 174 (160-188) |
| | | 2008 | 570 (547-595) | 454 (433-476) | 86.9 (77.9-96.8) | 50.7 (43.6-58.6) | 210 (195-225) |
| | | 2009 | 548 (525-572) | 427 (406-448) | 90.7 (81.7-100) | 51.8 (44.7-59.7) | 217 (203-232) |
| | | 2010 | 552 (530-576) | 447 (426-468) | 88.5 (79.7-98.0) | 36.8 (31.0-43.5) | 233 (218-248) |
| | | 2011 | 520 (499-543) | 415 (396-435) | 92.5 (83.6-102) | 33.4 (28.0-39.6) | 229 (215-244) |
| | | 2012 | 534 (513-557) | 435 (416-455) | 84.6 (76.3-93.6) | 30.3 (25.3-36.1) | 220 (206-235) |
| | | 2013 | 498 (478-519) | 408 (389-427) | 82.0 (74.0-90.6) | 22.9 (18.7-27.9) | 223 (210-238) |
| | | 2014 | 475 (456-495) | 397 (379-415) | 75.6 (68.0-83.8) | 18.5 (14.8-22.9) | 210 (198-224) |
| | | 2015 | 442 (423-460) | 361 (344-378) | 79.5 (71.9-87.7) | 19.9 (16.1-24.4) | 198 (185-210) |
| | | 2016 | 452 (434-471) | 379 (362-396) | 73.4 (66.3-81.2) | 14.7 (11.6-18.4) | 194 (182-206) |
| | | 2017 | 454 (437-473) | 380 (364-397) | 74.7 (67.7-82.3) | 13.3 (10.3-16.8) | 199 (187-211) |
| | | 2018 | 433 (416-451) | 367 (351-383) | 66.1 (59.6-73.2) | 14.8 (11.8-18.4) | 176 (165-187) |
| | | 2019 | 422 (405-439) | 356 (341-372) | 68.2 (61.7-75.3) | 12.1 (9.36-15.3) | 169 (158-180) |
| | | 2020 | 422 (406-440) | 360 (344-376) | 67.3 (60.8-74.3) | 10.7 (8.15-13.7) | 180 (169-192) |
| | | 2021 | 407 (390-423) | 344 (329-360) | 69.2 (62.7-76.3) | 8.30 (6.12-11.0) | 164 (154-175) |
| Women | | 2001 | 336 (318-355) | 257 (242-274) | 59.9 (52.5-68.2) | 31.2 (25.8-37.3) | 104 (94.4-115) |
| | | 2002 | 341 (323-360) | 264 (248-281) | 57.2 (49.9-65.2) | 32.0 (26.6-38.3) | 98.7 (89.0-109) |
| | | 2003 | 348 (330-367) | 275 (259-292) | 57.5 (50.2-65.5) | 27.7 (22.7-33.6) | 95.9 (86.4-106) |
| | | 2004 | 320 (302-338) | 247 (232-264) | 54.6 (47.6-62.5) | 26.1 (21.2-31.8) | 98.2 (88.6-109) |
| | | 2005 | 320 (302-338) | 246 (231-262) | 57.6 (50.4-65.5) | 27.2 (22.2-32.9) | 110 (99.5-121) |
| | | 2006 | 324 (306-342) | 251 (236-267) | 56.6 (49.5-64.4) | 28.8 (23.8-34.7) | 108 (98.1-119) |
| | | 2007 | 309 (292-327) | 237 (222-253) | 51.7 (44.9-59.2) | 32.8 (27.4-39.0) | 123 (113-135) |
| | | 2008 | 334 (317-353) | 262 (246-278) | 52.2 (45.6-59.7) | 31.0 (25.8-37.1) | 164 (152-177) |
| | | 2009 | 296 (279-313) | 234 (220-249) | 44.1 (38.0-51.0) | 25.8 (21.0-31.2) | 160 (149-173) |
| | | 2010 | 302 (286-319) | 241 (227-256) | 48.0 (41.8-55.0) | 22.3 (18.0-27.3) | 185 (172-198) |
| | | 2011 | 302 (286-318) | 239 (225-254) | 55.4 (48.7-62.7) | 17.5 (13.9-21.9) | 178 (166-191) |
| | | 2012 | 284 (269-300) | 230 (216-244) | 45.1 (39.2-51.6) | 17.4 (13.8-21.6) | 184 (172-197) |
| | | 2013 | 273 (258-288) | 213 (200-226) | 52.9 (46.6-59.8) | 17.1 (13.6-21.3) | 185 (173-197) |
| | | 2014 | 264 (250-279) | 209 (196-222) | 51.2 (45.2-58.0) | 13.5 (10.4-17.2) | 172 (160-184) |
| | | 2015 | 262 (248-276) | 211 (198-224) | 47.7 (41.9-54.0) | 12.1 (9.28-15.5) | 159 (148-171) |
| | | 2016 | 252 (238-266) | 202 (190-215) | 46.2 (40.6-52.4) | 11.3 (8.58-14.6) | 144 (134-154) |
| | | 2017 | 245 (232-259) | 199 (187-211) | 45.6 (40.2-51.6) | 9.32 (6.94-12.2) | 162 (151-173) |
| | | 2018 | 233 (221-246) | 191 (180-202) | 42.0 (36.8-47.6) | 7.59 (5.49-10.2) | 151 (141-161) |
| | | 2019 | 245 (232-258) | 203 (192-215) | 42.6 (37.5-48.2) | 6.38 (4.49-8.80) | 147 (137-157) |
| | | 2020 | 229 (217-242) | 186 (175-197) | 43.5 (38.3-49.2) | 7.84 (5.74-10.5) | 138 (128-148) |
| | | 2021 | 231 (219-243) | 187 (176-198) | 46.9 (41.6-52.8) | 5.07 (3.42-7.24) | 132 (123-142) |

| sex | age | year | Total | IS | ICH | US | TIA |
|---|---|---|---|---|---|---|---|
| Men | 75+ | 2001 | 2793 (2687-2904) | 1918 (1833-2008) | 339 (306-376) | 622 (568-681) | 531 (486-580) |
| | | 2002 | 2797 (2691-2908) | 1941 (1855-2031) | 328 (296-364) | 624 (570-684) | 560 (514-611) |
| | | 2003 | 2726 (2622-2835) | 1897 (1812-1985) | 317 (285-354) | 579 (528-635) | 549 (505-599) |
| | | 2004 | 2634 (2533-2739) | 1874 (1791-1961) | 311 (279-347) | 538 (490-591) | 539 (494-588) |
| | | 2005 | 2637 (2537-2742) | 1871 (1789-1957) | 344 (310-381) | 502 (455-554) | 513 (471-559) |
| | | 2006 | 2502 (2406-2602) | 1774 (1696-1857) | 329 (297-365) | 459 (415-508) | 570 (525-620) |
| | | 2007 | 2447 (2353-2545) | 1751 (1673-1832) | 322 (291-357) | 449 (407-496) | 583 (538-632) |
| | | 2008 | 2387 (2295-2483) | 1667 (1592-1746) | 354 (321-391) | 451 (409-497) | 623 (577-673) |
| | | 2009 | 2307 (2217-2401) | 1656 (1581-1735) | 329 (298-364) | 395 (356-437) | 600 (555-648) |
| | | 2010 | 2264 (2176-2357) | 1645 (1570-1723) | 341 (310-376) | 359 (323-399) | 674 (627-725) |
| | | 2011 | 2210 (2124-2299) | 1616 (1544-1692) | 338 (307-372) | 322 (288-359) | 634 (588-683) |
| | | 2012 | 2065 (1983-2151) | 1525 (1455-1598) | 316 (286-349) | 270 (240-305) | 631 (586-679) |
| | | 2013 | 1961 (1882-2044) | 1436 (1369-1506) | 343 (312-377) | 255 (226-288) | 632 (587-681) |
| | | 2014 | 1909 (1833-1988) | 1402 (1338-1470) | 351 (320-384) | 238 (210-270) | 618 (574-665) |
| | | 2015 | 1852 (1777-1930) | 1399 (1335-1466) | 319 (290-351) | 204 (178-232) | 588 (546-634) |
| | | 2016 | 1850 (1776-1926) | 1372 (1309-1437) | 335 (305-366) | 209 (184-237) | 554 (514-597) |
| | | 2017 | 1768 (1697-1841) | 1352 (1291-1416) | 310 (282-341) | 169 (147-194) | 556 (516-598) |
| | | 2018 | 1743 (1674-1815) | 1355 (1295-1418) | 303 (276-332) | 142 (123-165) | 519 (481-560) |
| | | 2019 | 1795 (1726-1867) | 1399 (1339-1462) | 323 (295-352) | 135 (116-157) | 543 (505-583) |
| | | 2020 | 1700 (1635-1768) | 1317 (1260-1377) | 315 (288-343) | 126 (108-147) | 542 (506-582) |
| | | 2021 | 1660 (1597-1725) | 1333 (1278-1392) | 293 (268-320) | 89.5 (74.8-107) | 515 (480-553) |
| Women | | 2001 | 2193 (2128-2259) | 1454 (1402-1507) | 266 (244-288) | 544 (511-578) | 438 (410-468) |
| | | 2002 | 2236 (2172-2303) | 1483 (1431-1537) | 253 (232-275) | 563 (530-597) | 444 (416-474) |
| | | 2003 | 2216 (2151-2281) | 1452 (1400-1505) | 269 (248-291) | 549 (517-583) | 433 (405-463) |
| | | 2004 | 2086 (2023-2149) | 1413 (1362-1465) | 232 (212-253) | 496 (465-527) | 413 (385-442) |
| | | 2005 | 1967 (1907-2029) | 1340 (1291-1391) | 236 (216-257) | 446 (418-476) | 412 (384-441) |
| | | 2006 | 2027 (1966-2089) | 1371 (1322-1422) | 251 (231-273) | 454 (426-485) | 435 (407-464) |
| | | 2007 | 1870 (1812-1930) | 1291 (1243-1341) | 227 (207-248) | 411 (384-440) | 478 (448-509) |
| | | 2008 | 1900 (1841-1960) | 1306 (1257-1355) | 239 (219-260) | 407 (381-435) | 526 (494-558) |
| | | 2009 | 1740 (1684-1797) | 1189 (1143-1237) | 228 (208-249) | 375 (349-401) | 533 (501-566) |
| | | 2010 | 1717 (1661-1774) | 1185 (1139-1233) | 220 (201-241) | 352 (328-378) | 559 (527-593) |
| | | 2011 | 1701 (1646-1758) | 1209 (1162-1257) | 223 (203-244) | 316 (293-340) | 554 (522-588) |
| | | 2012 | 1655 (1601-1711) | 1167 (1122-1215) | 237 (217-258) | 305 (282-329) | 581 (547-615) |
| | | 2013 | 1504 (1452-1558) | 1089 (1044-1134) | 235 (215-257) | 227 (208-248) | 553 (521-587) |
| | | 2014 | 1494 (1443-1547) | 1073 (1029-1118) | 237 (217-259) | 237 (218-258) | 526 (495-559) |
| | | 2015 | 1360 (1311-1410) | 981 (940-1024) | 215 (196-235) | 209 (191-228) | 481 (452-513) |
| | | 2016 | 1449 (1399-1501) | 1041 (998-1085) | 249 (229-271) | 200 (183-219) | 490 (460-521) |
| | | 2017 | 1356 (1307-1406) | 1003 (961-1046) | 234 (214-255) | 170 (154-188) | 496 (466-528) |
| | | 2018 | 1374 (1326-1424) | 1020 (978-1064) | 244 (224-265) | 163 (147-180) | 475 (446-506) |
| | | 2019 | 1340 (1292-1389) | 1000 (959-1042) | 248 (228-269) | 133 (119-149) | 472 (443-502) |
| | | 2020 | 1264 (1218-1311) | 968 (928-1010) | 220 (201-239) | 121 (107-135) | 440 (413-469) |
| | | 2021 | 1303 (1257-1350) | 1012 (972-1053) | 245 (226-266) | 90.6 (79.2-103) | 445 (418-474) |

## Figures

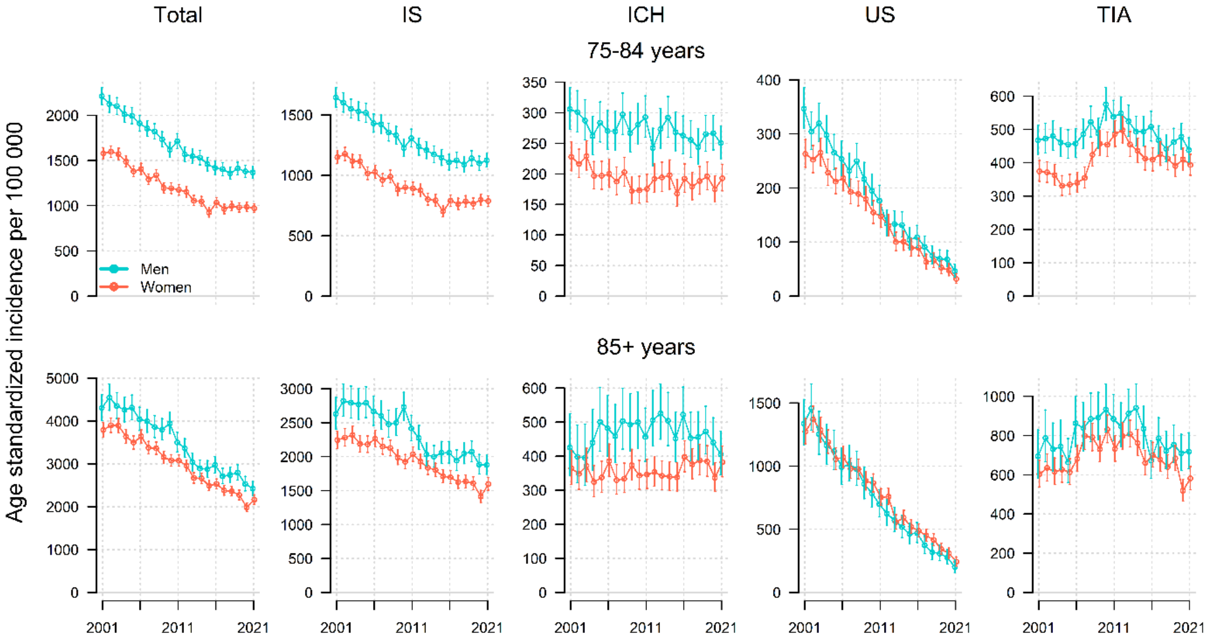


Figure S1. Age-standardized incidence per 100,000 population by sex and age groups for the elderly population for total stroke, ischemic stroke, intracerebral hemorrhage, unspecified stroke and TIA in Norway, 2001 to 2021. Total= total stroke, IS= ischemic stroke, ICH= intracerebral hemorrhage, US= Unspecified stroke, pyrs= personyears.

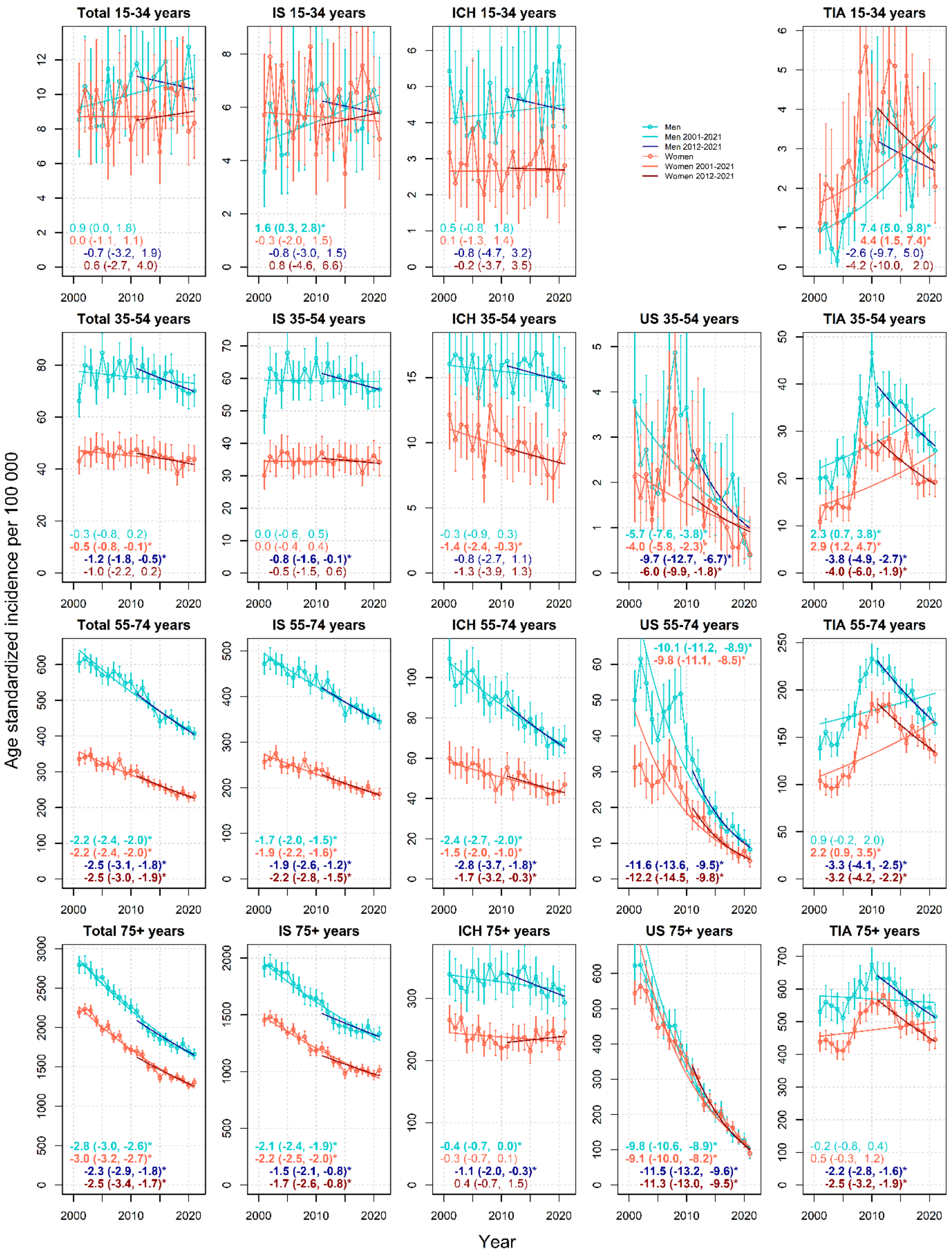
Total 15-34 years
0.9 (0.0, 1.8)
0.0 (-1.1, 1.1)
-0.7 (-3.2, 1.9)
0.6 (-2.7, 4.0)
IS 15-34 years
1.6 (0.3, 2.8)*
-0.3 (-2.0, 1.5)
-0.8 (-3.0, 1.5)
0.8 (-4.6, 6.6)
ICH 15-34 years
0.5 (-0.8, 1.8)
0.1 (-1.3, 1.4)
-0.8 (-4.7, 3.2)
-0.2 (-3.7, 3.5)
Men
Men 2001-2021
Men 2012-2021
Women
Women 2001-2021
Women 2012-2021
TIA 15-34 years
7.4 (5.0, 9.8)*
4.4 (1.5, 7.4)*
-2.6 (-9.7, 5.0)
-4.2 (-10.0, 2.0)
Total 35-54 years
-0.3 (-0.8, 0.2)
-0.5 (-0.8, -0.1)*
-1.2 (-1.8, -0.5)*
-1.0 (-2.2, 0.2)
IS 35-54 years
0.0 (-0.6, 0.5)
0.0 (-0.4, 0.4)
-0.8 (-1.6, -0.1)*
-0.5 (-1.5, 0.6)
ICH 35-54 years
-0.3 (-0.9, 0.3)
-1.4 (-2.4, -0.3)*
-0.8 (-2.7, 1.1)
-1.3 (-3.9, 1.3)
US 35-54 years
-5.7 (-7.6, -3.8)*
-4.0 (-5.8, -2.3)*
-9.7 (-12.7, -6.7)*
-6.0 (-9.9, -1.8)*
TIA 35-54 years
2.3 (0.7, 3.8)*
2.9 (1.2, 4.7)*
-3.8 (-4.9, -2.7)*
-4.0 (-6.0, -1.9)*
Total 55-74 years
-2.2 (-2.4, -2.0)*
-2.2 (-2.4, -2.0)*
-2.5 (-3.1, -1.8)*
-2.5 (-3.0, -1.9)*
IS 55-74 years
-1.7 (-2.0, -1.5)*
-1.9 (-2.2, -1.6)*
-1.9 (-2.6, -1.2)*
-2.2 (-2.8, -1.5)*
ICH 55-74 years
-2.4 (-2.7, -2.0)*
-1.5 (-2.0, -1.0)*
-2.8 (-3.7, -1.8)*
-1.7 (-3.2, -0.3)*
US 55-74 years
-10.1 (-11.2, -8.9)*
-9.8 (-11.1, -8.5)*
-11.6 (-13.6, -9.5)*
-12.2 (-14.5, -9.8)*
TIA 55-74 years
0.9 (-0.2, 2.0)
2.2 (0.9, 3.5)*
-3.3 (-4.1, -2.5)*
-3.2 (-4.2, -2.2)*
Total 75+ years
-2.8 (-3.0, -2.6)*
-3.0 (-3.2, -2.7)*
-2.3 (-2.9, -1.8)*
-2.5 (-3.4, -1.7)*
IS 75+ years
-2.1 (-2.4, -1.9)*
-2.2 (-2.5, -2.0)*
-1.5 (-2.1, -0.8)*
-1.7 (-2.6, -0.8)*
ICH 75+ years
-0.4 (-0.7, 0.0)*
-0.3 (-0.7, 0.1)
-1.1 (-2.0, -0.3)*
0.4 (-0.7, 1.5)
US 75+ years
-9.8 (-10.6, -8.9)*
-9.1 (-10.0, -8.2)*
-11.5 (-13.2, -9.6)*
-11.3 (-13.0, -9.5)*
TIA 75+ years
-0.2 (-0.8, 0.4)
0.5 (-0.3, 1.2)
-2.2 (-2.8, -1.6)*
-2.5 (-3.2, -1.9)*
Age standardized incidence per 100 000
Year
2000
2010
2020

Figure S2. Age-standardized annual incidence per 100,000 population with 95% confidence intervals (CIs) by sex and age group for total stroke (Total), ischemic stroke (IS), intracerebral hemorrhage (ICH), unspecified stroke (US) and transient ischaemic attack (TIA) in Norway, 2001-2021. The trendlines represent predicted IRs from the linear regressions log(IR) = $\alpha + \beta$ • year over the whole period (light trendlines) and the last 10 years (dark trendlines). The numbers represent annual increase as estimated from the regressions, i.e. (exp($\beta$)-1) •100, with 95% confidence intervals. * $p < 0.05$.

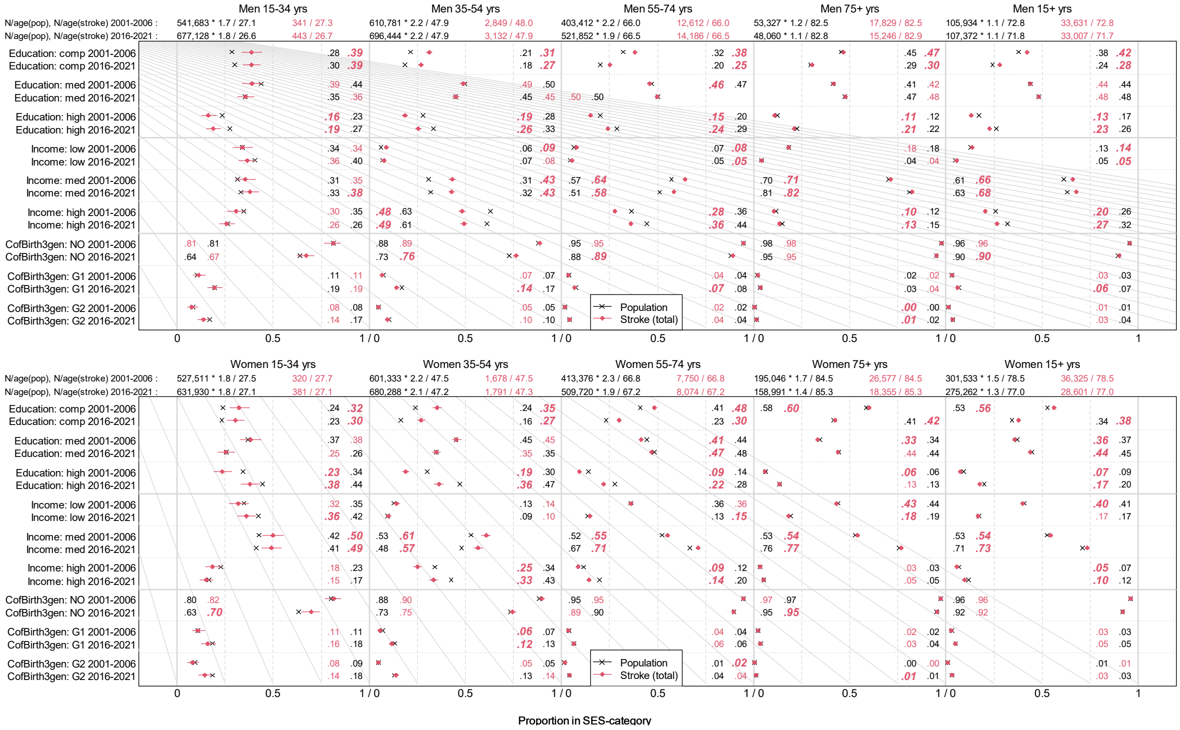

Figure S3. Education, income and country background (CofBirth3gen) for incident total stroke patients and the (down-sampled*) general Norwegian population 2001-2006 and 2016-2021, by sex and age group.

In each age group the proportion of the stroke patients with low, medium and high education; low, medium and high income; and Norwegian, western, and non-western background are shown in red for the stroke population and black for the general population, with 95% confidence intervals (too narrow to be visible for the general population). The red numbers at top indicate for each 5 year period the total number of stroke patients and their mean age at stroke. The black numbers for the general population indicate the number of unique individuals times the average number of years each individual contributed (the same person may contribute all five years, but due to the random down-sampling, the average is lower). Large red bold italic numbers inside the figure indicate non-overlapping confidence intervals.

*Down-sampling of general population: The age distribution of the stroke population is skewed within each 20 year age group, with an overweight of older individuals. The general population was down-sampled by excluding randomly selected younger individuals so that the proportion of the population that were in each one year age group was similar in the general population as in the general population in each calendar year.

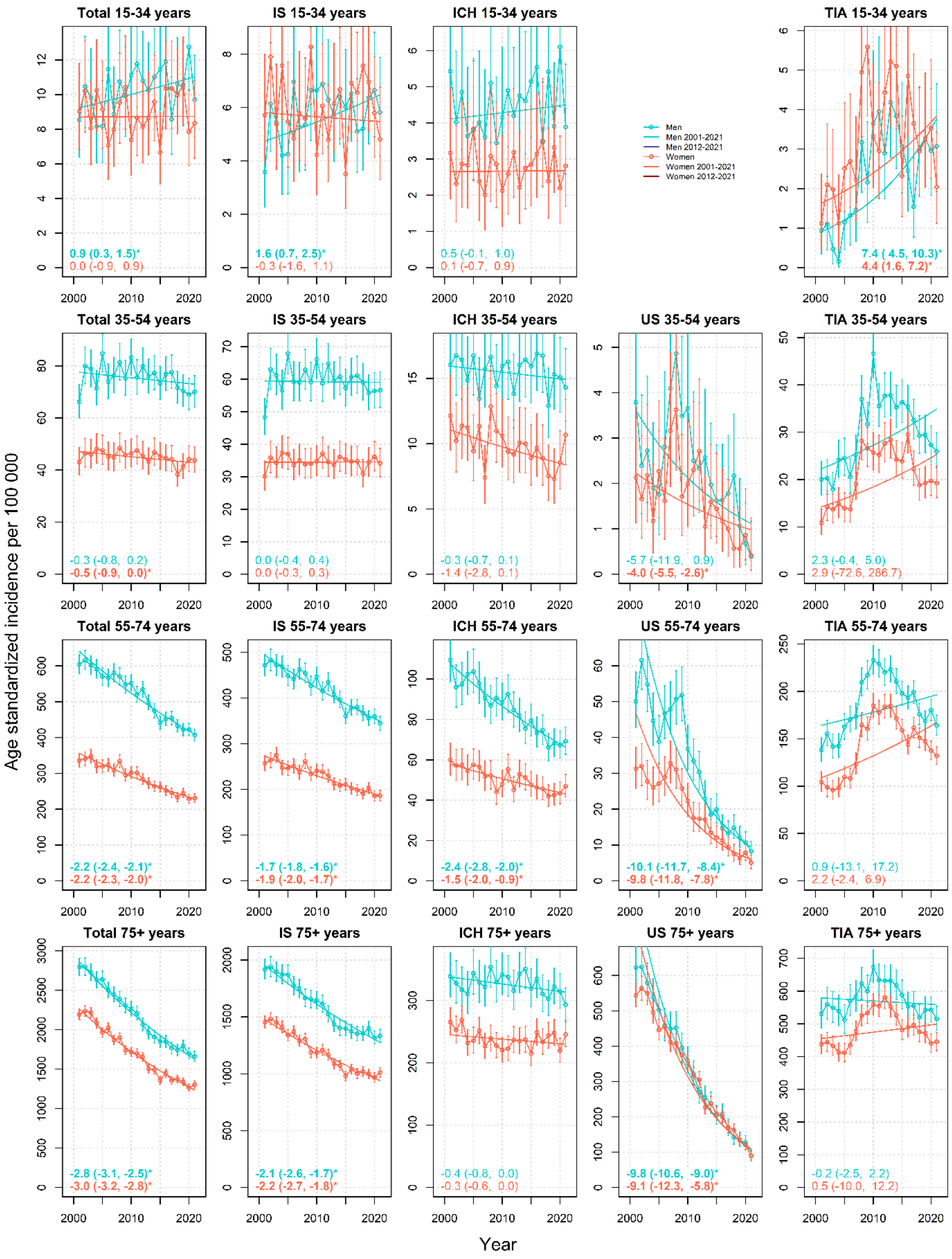


Figure S4. Sensitivity analysis. Age-standardized annual incidence per 100,000 population with 95% confidence intervals (CIs) by sex and age group for total stroke (Total), ischemic stroke (IS), intracerebral hemorrhage (ICH), unspecified stroke (US) and transient ischemic attack (TIA) in Norway, 2001-2021. The 95% confidence intervals for the annual percentage change are calculated by taking the autocorrelation in the residuals between adjacent years into account. The trendlines represent predicted IRs from the linear regressions log(IR) = α+β • year over the whole period.

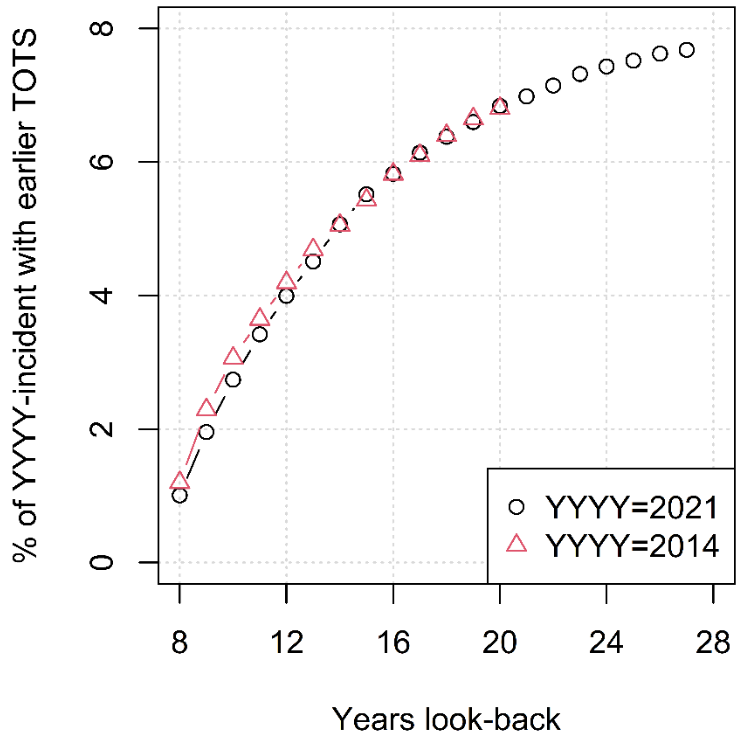


Figure S5. Lookback period and estimated percentage with previous stroke classified as incident stroke. Circle represent incident stroke in 2021 and triangle represent incident stroke in 2014. TOTS= total stroke.
